# Brain Activity in Response to Trauma-specific, Negative, and Neutral Stimuli. A fMRI Study of Recent Road Traffic Accident Survivors

**DOI:** 10.3389/fpsyg.2016.01173

**Published:** 2016-08-05

**Authors:** Andre S. Nilsen, Ines Blix, Siri Leknes, Øivind Ekeberg, Laila Skogstad, Tor Endestad, Bjørn C. Østberg, Trond Heir

**Affiliations:** ^1^Norwegian Centre for Violence and Traumatic Stress StudiesOslo, Norway; ^2^Center for the Study of Human Cognition, Department of Psychology, University of OsloOslo, Norway; ^3^The Intervention Centre, Oslo University HospitalOslo, Norway; ^4^Division of Mental Health and Addiction, Oslo University HospitalOslo, Norway; ^5^Department of Behavioral Sciences in Medicine, Institute of Basic Medical Sciences, Faculty of Medicine, University of OsloOslo, Norway; ^6^Research and Development, Department of Acute Medicine, Oslo University HospitalOslo, Norway; ^7^Institute of Psychiatry, King’s College LondonLondon, UK; ^8^Institute of Clinical Medicine, Faculty of Medicine, University of OsloOslo, Norway

**Keywords:** traumatic stress, trauma-exposure, functional connectivity, attentional bias, visual cortex, amygdala, road traffic accident, occipital cortex

## Abstract

Most studies of neuro-functional patterns in trauma-exposed individuals have been conducted considerable time after the traumatic event. Hence little is known about neuro-functional processing shortly after trauma-exposure. We investigated brain activity patterns in response to trauma reminders as well as neutral and negative stimuli in individuals who had recently (within 3 weeks) been involved in a road traffic accident (RTA). Twenty-three RTA survivors and 17 non-trauma-exposed healthy controls (HCs) underwent functional MRI while viewing Trauma-specific, Negative, and Neutral pictures. Data were analyzed from four *a priori* regions of interest, including bilateral amygdala, subcallosal cortex, and medial prefrontal cortex. In addition, we performed a whole brain analysis and functional connectivity analysis during stimulus presentation. For both groups, Negative stimuli elicited more activity in the amygdala bilaterally than did Neutral and Trauma-specific stimuli. The whole brain analysis revealed higher activation in sensory processing related areas (bilateral occipital and temporal cortices and thalamus) as well as frontal and superior parietal areas, for the RTA group compared to HC, for Trauma-specific stimuli contrasted with Neutral stimuli. We also observed higher functional connectivity for Trauma-specific stimuli, between bilateral amygdala and somatosensory areas, for the RTA group compared to controls, when contrasted with Neutral stimuli. We argue that these results might indicate an attentional sensory processing bias toward Trauma-specific stimuli for trauma exposed individuals, a result in line with findings from the post-traumatic stress disorder literature.

## Introduction

After experiencing a traumatic event, some individuals develop post-traumatic stress reactions, such as hyper-arousal, intrusive thoughts and memories, avoidance of trauma reminding stimuli, and trauma related memories or thoughts (APA, 2000). These symptoms can persist for days, weeks, or years. Although, some individuals develop long-lasting symptoms of post-traumatic stress disorder (PTSD), for most individuals these symptoms subside gradually over time (McFarlane, 1997).

Lines of research have focused on neuro-functional alterations associated with trauma-exposure, and PTSD in particular. The leading model, ‘the fear circuitry model of PTSD’ proposes that increased amygdala activity in response to threatening stimuli, and simultaneous decreased activity in prefrontal areas of the cortex that normally suppress the amygdala, underlies intrusive memories and hyper-arousal symptoms (Rauch et al., 2006). In line with the fear circuitry model, recent meta-analyses focusing on neuro-functional patterns associated with PTSD, have demonstrated hyperactive amygdala and hypoactive prefrontal regions (Hayes et al., 2012; Patel et al., 2012), as well as hyperactive hippocampus (Patel et al., 2012). Furthermore, abnormal functioning of the hippocampus has been suggested to reflect alterations in memory associated with PTSD (Shin et al., 2006). Recently, attention has also been directed toward alterations in dorsal anterior cingulate cortex (dCCA; Hughes and Shin, 2011; Hayes et al., 2012), an area where increased activity is associated with heightened threat evaluation and appraisal (Hayes et al., 2012). Moreover, recent studies have shown that PTSD is associated with altered functional connectivity between the amygdala, and medial prefrontal cortex (mPFC), insula, and dCCA (Gilboa et al., 2012; Stevens et al., 2013), suggesting an interplay between prefrontal regions and limbic structures.

A pertinent question concerns whether the neuro-functional alterations associated with trauma-exposure and post-traumatic stress reflects general alterations in activation for processing of negative or threatening emotional material, or if alterations are specifically connected to trauma-related material. The results have been inconsistent with some studies demonstrating neuro-functional alterations specifically connected to trauma-related or threatening material (Rauch et al., 1996; Liberzon et al., 1999; Hendler et al., 2003; Shin et al., 2004; Protopopescu et al., 2005), while other studies have reported general alterations in activation irrespective of valence or trauma relevance (Hendler et al., 2003; Brunetti et al., 2010).

The majority of studies on neuro-functional patterns in the aftermath of trauma have focused on PTSD, and less attention has been directed toward the effects of trauma-exposure *per se*. Knowledge about brain activity associated with trauma-exposure *per se* is vital for understanding post-trauma cognition and emotional processing, and might provide important insights into the development of PTSD. Furthermore, most studies have either compared individuals with PTSD with trauma-exposed controls or with non-trauma-exposed controls, and as pointed out by Stark et al. (2015), the control group is used a baseline to PTSD and different control groups might result in different neuro-functional patterns. Therefore, there is a need for more knowledge on how trauma-exposed groups may differ from non-trauma-exposed groups, and to understand the effects of trauma exposure *per se*.

A few meta-analyses have classified studies according to the trauma-status of control group (Patel et al., 2012; Stark et al., 2015). Patel et al. (2012) reported that neuro-functional alterations are observed in trauma-exposed individuals without a diagnosis of PTSD. For example, trauma-exposed individuals, compared to non-trauma-exposed controls, showed greater activity in prefrontal regions. This might suggest that prefrontal areas are implicated in coping and resilience in the aftermath of trauma. As for amygdala activation, the meta-analysis showed that hyperactivity was *only* found when PTSD participants were compared to non-trauma-exposed controls (Patel et al., 2012). Thus, contrary to a perspective suggesting that a hyperactive amygdala is a marker for PTSD, Patel et al. (2012) propose that trauma exposure itself can lead to a general increase in amygdala responsivity, reflecting overall increased stimuli threat appraisal.

Additionally, a recent meta-analysis by Stark et al. (2015), reported different patterns when comparing PTSD with trauma-exposed vs. non-trauma-exposed control groups. When compared to trauma-exposed controls, PTSD participants showed differential activation in regions in the basal ganglia, among others. However, when PTSD groups were compared with non-trauma-exposed controls the results revealed a differential pattern of activation in the right anterior insula, precuneus, cingulate and bilateral orbitofrontal cortex, a pattern that did not overlap with results when PTSD groups were compared to trauma-exposed controls.

Taken together, these findings suggest that some of the neuro-functional alterations seen in PTSD are not disorder specific but rather characteristic for people who have been exposed to a traumatic event, such as a hyperactive amygdala. However, medial prefrontal activity might reflect coping and resilience following trauma exposure, and a failure to do so in PTSD. Thus, as suggested by Patel et al. (2012) and Stark et al. (2015), there might be distinct neuro-functional patterns associated with trauma-exposure *per se*. However, these meta-analyses did not compare the two types of control groups directly. Hence, as pointed by Stark et al. (2015) there is a need for studies that directly investigate how neuro-functional patterns in trauma-exposed groups may differ from non-trauma-exposed groups.

In the present study we aimed to follow up on these findings, and focus on patterns of brain activity in individuals who have recently been exposed to trauma. The main aim in the present study was to investigate neuro-functional activity in individuals who had recently been admitted to the hospital following a road traffic accident (RTA). More specifically, we wanted to investigate whether similar neuro-functional patterns as those observed in previous PTSD studies (as described by the fear-circuitry model above) can also be found in recently trauma-exposed individuals, that is in the time before a PTSD diagnosis can be given at 1 month post-trauma (APA, 2000). A second aim was to address whether possible neuro-functional differences in recently trauma-exposed individuals are specifically connected to processing of trauma-relevant material, or general threatening material. To achieve this, participants were presented to visual stimuli material consisting of trauma reminders (Trauma-specific), threatening (Negative violence), and everyday objects (Neutral).

In line with previous literature we hypothesized hyperactivity in amygdala for Trauma-specific material. As for medial prefrontal regions, we had no specific hypothesis for the directionality of any observed effects. Further, we aimed to explore the functional coupling between amygdala and other cortical regions during Trauma-specific stimuli presentation.

## Materials and Methods

### Inclusion and Exclusion Criteria

Inclusion criteria for the hospitalized RTA survivors were; age between 18 and 60 years (for motorcycle and bicycle accidents; speed, injury, and/or contact with car); admitted to hospital with injuries caused by RTA; trauma team summoned; discharged home from hospital, signed informed consent, and participated, all within 3 weeks of the accident. Exclusion criteria were; reduced consciousness on admission (Glasgow Coma Scale <14), without a permanent address, non-Norwegian speaker, known brain trauma, MR incompatible implants (pacemaker, neural stimulator, etc.), immobilized or amnesia at study time.

### Participants

Twenty-seven hospitalized RTA survivors and 18 healthy age and education matched healthy controls (HC) agreed to participate in the study. Four RTA participants were excluded due to concussion (1), pain (2), claustrophobia (1), and one HC was later excluded due to an incidental pathological finding. This left a total sample of 23 RTA participants (Males = 18, Age = 40.2, *SD* = 12.5) and 17 HC participants (Males = 12, Age = 37.1, *SD* = 9.6).

A member of the research team screened all patient records after admission to the emergency department at Oslo University Hospital (OUS) or Akershus Hospital (AHUS). Eligible patients were contacted, informed about the study and asked to participate. A checklist for eligibility assessment was used, where all inclusion criteria and none of the exclusion criteria had to be met. The inclusion of patients and testing was done within 21 days after hospital admission. The HCs were recruited among blood donors at Oslo University Hospital’s Blood Centre.

The study was approved by the regional committee for medical and health research ethics. All participants were informed about the purpose and content of the study and given the opportunity to withdraw.

### Measures

Post-traumatic check list-specific (PCL-S; Weathers et al., 1994), translated to Norwegian (Hem et al., 2012), was used to assess PTSD symptoms as described in DSM-IV. The participants were asked to indicate on a five-point scale to which extent they had been bothered by 17 symptoms since the accident. A total symptom severity score (range = 17–85) was calculated by summing up the scores from each of the 17 items. For the participants in the RTA group the items in the PCL-S were specifically linked to the traffic accident. The HC participants were asked to refer to the most personally experienced stressful negative event they could think of. The PCL-S commonly operates with a cut-off score of 50, indicating possible future diagnosis of PTSD (Blanchard et al., 1996).

### Stimuli and Design

Picture stimuli were obtained from Anke Ehlers and Birgit Kleim and the material was previously piloted by Ehlers and colleagues at King’s College (personal communication, 2011); healthy participants (*n* = 25) rated three blocks of pictures (350 each) for arousal and emotional valence. The Trauma-specific stimuli consisted of RTA photos and the Negative stimuli consisted of violence photos, while the Neutral stimuli consisted of everyday pictures (ironing, TV, clothes, house, tennis, etc.). The Negative and Trauma-specific pictures were matched in terms of emotional valence and arousal. All lists and blocks were also matched in terms of valence and arousal. The pictures were matched across the three conditions for luminance and size.

### Experimental Task

Stimuli were shown on a screen located ~100 cm behind the scanner bore, and viewed via a mirror mounted on the head coil. The pictures were presented in single condition blocks lasting 16 s, with eight pictures of one condition in each block, with each picture presented for 2 s. In between blocks was a fixation cross for 8 s. There was a total of 30 blocks, 10 for each condition (Trauma-specific, Negative, and Neutral). To control for wakefulness during the task, participants were instructed to press a button when a target stimulus (mushroom, 2 s) appeared within a block (0, 1, or 2 targets per block, balanced between stimuli conditions). Inclusion in the analysis was set at >75% accuracy for this attention task. To control for order and stimuli specific effects, two different lists of pictures were used, and the order of blocks and the stimuli within each block was pseudorandomized in two different sequences, resulting in a total of four different experimental stimuli lists. The task lasted for 12 min.

### fMRI Data Acquisition

Scanning was performed on two scanners; a three Tesla Philips Achieva whole body MR scanner equipped with an eight-channel Philips SENSE head coil (Philips Medical Systems, Best, the Netherlands).

The functional data were acquired with a blood oxygen level dependent (BOLD) sensitive T2* echo-planar imaging sequence (TR 2.208 s, TE 30 ms, FOV 240 × 240 × 126, flip angle 80°, serial acquisition, SENSE 2.3) with 42 slices and a voxel size of 3 mm × 3 mm × 3 mm, and reconstructed into a 80 × 80 × 42 matrix. One session consisted of 335 volumes, and lasted 12 min and 40 s. The first five volumes were discarded to allow for MR signal equilibrium. For oﬄine registration of the functional data, an anatomical T1 weighted image was acquired (TR 4.5 ms, TE 2.2 ms; FOV 256 × 256 × 204, flip angle 8°, SENSE 1.2) with voxel size 1 × 1 × 1.2 and 170 slices.

### Procedure

Before scanning, the participants were informed about the project, signed an informed consent form and were screened for MR compatibility. Participants were first scanned with two calibration scans, followed by the fMRI experimental task, lasting 12 min, finishing with a structural image. After the scanning session, participants completed the PCL-S questionnaire.

### Analyses

All fMRI data were preprocessed and analyzed using FSL (FMRIB’s Software Library^1^, version 6.00). The following pre-processing steps were performed; motion correction using FMRIB’s Linear Image Registration Tool (MCFLIRT; Jenkinson and Smith, 2001); brain extraction using Brain Extraction Tool (BET; Smith, 2002); spatial smoothing using a Gaussian kernel with 5 mm fill width at half maximum; grand-mean intensity normalization of the entire 4D dataset by a single multiplicative factor; high pass temporal filtering (Gaussian-weighted least-squares straight line fitting, sigma = 100 s).

Registration of functional images to Montreal Neurological Institute (MNI) standard space [12 degrees of freedom (DOF)] through the structural T1 weighed image (12 DOF), was performed using FMRIB’s Linear Image Registration Tool (FLIRT; Jenkinson and Smith, 2001; Jenkinson et al., 2002). Both brain extraction and registration were subjected to visual quality control.

The design matrix of the general linear model (GLM) contained three explanatory variables (EVs) of interest: Neutral, Negative, and Trauma-specific (see Stimuli and Design), and one EV of non-interest: target. The EV’s were modeled as boxcars spanning each block type, and convolved with a double gamma HRF. Each participant’s sessions were analyzed with a first level analysis with in total four main contrasts; Trauma-specific vs. Neutral, and vs. Negative; and Negative vs. Trauma-specific, and vs. Neutral. Time-series statistical analysis was carried out using FMRIB’s Improved Linear Model (FILM) with local autocorrelation correction (Woolrich et al., 2001).

To explore overall effects of traumatic experiences, a whole brain analysis was performed. The resulting COPE images from the individual analysis were combined in a higher level local mixed effect analysis using FLAME stage 1 plus 2 with automatic outlier detection (Beckmann et al., 2003; Woolrich et al., 2004; Woolrich, 2008). Z (Gaussianized T/F) statistic images were thresholded using clusters determined by *Z* > 2.3 and a corrected cluster significance threshold of *p* = 0.05 (Worsley, 2001).

To investigate the predicted hyper activation of bilateral amygdala and activation levels of mPFC, four regions of interest (ROI) were created from the Harvard-Oxford cortical and subcortical atlas^2^; left and right amygdala, subcallosal cortex, and frontal medial cortex. The last two ROIs were chosen due to their implication in several earlier studies (Bremner et al., 1999; Rauch et al., 2003; Shin et al., 2006; Stark et al., 2015). Each ROI were set at a probability threshold of 50–100% and registered to each participant using the inverted transformation matrices from the registration. Mean BOLD activation levels for Neutral, Negative, and Trauma-specific stimuli, in each ROI, were extracted. Next, each ROI’s mean activation levels were analyzed in Statistical Package for Social Sciences (SPSSs). Due to satisfying threshold for normality (Shapiro–Wilk > 0.05) in 22/24 distributions, and close (0.05 > *p* > 0.04) in 2/24, we used a repeated measures analysis of variance (ANOVA) with stimuli type (Neutral, Trauma-specific, Negative) as within subject variable, and group (RTA, HC) as between subject variable.

To investigate a relationship between PCL scores and measured ROI BOLD signal change, a correlation analysis was performed for the sample as a whole and for the RTA and HC groups separately, with PCL scores as predictors for BOLD signal change in each of the four selected ROIs for each of the three stimuli conditions. Due to PCL scores violating the assumption of normality, Spearman’s Rank-Order Correlation was used.

To explore functional connectivity a psychophysiological interaction (PPI) analysis was modeled as an interaction between the demeaned time series of bilateral amygdala (Harvard-Oxford Sub-cortical atlas, 80–100% threshold) and the centered Neutral, Negative, and Trauma-specific stimulus time series (run separately, against implicit baseline), as well as the main contrasts Trauma-Specific vs. Neutral, and Negative, and Negative vs. Neutral, and Trauma-Specific. Only the four main contrasts will be discussed, while the contrasts including implicit baseline are reported only. The PPI analysis employed the same cluster thresholds and corrections as the whole-brain analysis. The left and right amygdala was chosen due to their importance in earlier PTSD studies (Patel et al., 2012), however, due to no specific hypothesis regarding the directionality or localization of functional connectivity changes, and for a more robust time series estimation, we chose a bilateral amygdala ROI. The resulting whole brain cluster table is reported.

## Results

The RTA group had significantly higher PCL scores than the HC group (*U* = 100.5, *p* = 0.016), however, only four participants exceeded a preliminary possible PTSD cutoff score of 50. Participants did not differ in terms of age, *t*(38) = 0.819, *p* = 0.418. See **Table 1**.

**Table 1:** Mean participant characteristics.

	RTA	HC
Number	23	17
Age (mean)	40.2 (12.5)	37.1 (9.6)
Number of females	5	5
PCL score (mean)*	31.8 (14.7)	22.4 (6.3)

Standard deviation in brackets. RTA, road traffic accident (Trauma) group; HC, healthy controls; PCL, post-traumatic check list. *p < 0.05.

None of the participants who completed the scanning session were excluded based on lack of attention in the affective stimuli task (<75% accuracy) nor due to excessive head motion (>0.5 mm absolute displacement).

For the whole brain analysis (see **Table 2**) we investigated between group differences in four main contrasts of interest: Trauma-specific vs. Negative and Neutral stimuli, and Negative stimuli vs. Trauma-specific and Neutral stimuli.

**Table 2:** Whole brain analysis results for RTA group vs. HC-group.

Voxels	*p*	*Z*	*x*	*y*	*z*	Side		Peak location
**Trauma-specific > Neutral, RTA > HC**								
4602	<0.00001	4,42	36	–64	36	R	35%	L. Occipital C.
		4,28	48	–50	–18	R	52%	I. Temporal G.
							28%	Temporal Occipital Fusiform C.
		4,17	32	–66	44	R	69%	L. Occipital C.
		3,98	52	–52	–16	R	58%	I. Temporal G.
1561	<0.00001	3,68	–34	–74	52	L	57%	L. Occipital C.
		3,61	–38	–64	28	L	15%	L. Occipital C.
							10%	Angular G.
		3,56	–24	–56	56	L	36%	S. Parietal Lobe
		3,5	–46	–72	34	L	78%	L. Occipital C.
981	0.000156	3,62	–4	–34	–18		100%	Brain-Stem
		3,6	4	–6	6	R	99%	Thalamus
		3,4	8	–42	–2	R	14%	Lingual G.
		3,38	8	–46	2	R	41%	Cingulate G.
		3,34	–4	–32	–2		69%	Brain-Stem
602	0.00615	3,61	–46	–54	–18	L	46%	I. Temporal G.
							35%	Temporal Occipital Fusiform C.
		3,53	–30	–36	–22	L	80%	Temporal Fusiform C.
		3,44	–34	–44	–22	L	48%	Temporal Fusiform C.
							39%	Temporal Occipital Fusiform C.
		3,36	–58	–72	–8	L	15%	L. Occipital C.
580	0.00776	3,96	54	28	28	R	41%	M. Frontal G.
		3,72	52	34	10	R	38%	I. Frontal G.
		3,46	52	38	12	R	58%	Frontal Pole
524	0.0142	3,64	–4	–56	20	L	58%	Precuneous C.
		3,24	–6	–42	4	L	21%	Cingulate G.
							11%	Hippocampus
		3,2	0	–64	28		84%	Precuneous C.
**Trauma-specific>Negative, RTA>HC**								
1678	<0.00001	3,74	28	–72	42	R	70%	L. Occipital C.
553	0.00743	4,34	28	–34	–16	R	52%	Parahippocampal G.
							26%	Temporal Fusiform C.
		3,48	8	–42	0	R	21%	Cingulate G.
							13%	Lingual G.
		3,35	30	–42	–14	R	51%	Temporal Occipital Fusiform C.
							10%	Temporal Fusiform C.
491	0.0152	4,34	48	–44	–12	R	29%	I. Temporal G.
		3,7	54	–48	–18	R	63%	I. Temporal G.
		3,39	58	–62	–12	R	38%	L. Occipital C.
		3,38	48	–50	–18	R	52%	I. Temporal G.
							28%	Temporal Occipital Fusiform C.
		3,33	58	–48	–12	R	38%	I. Temporal G.
							26%	M. Temporal G.

*Whole brain contrasts of clusters surviving corrections and threshold. Peak coordinates are in MNI space (mm). Z scores are reported for each cluster peak. Cluster extension: peaks above 10% of ROIs as defined by the Harvard Cortical and Subcortical Probability Atlas (http://fsl.fmrib.ox.ac.uk/fsl/fslwiki/Atlases) are reported. L, lateral; C, cortex; G, gyrus; I, inferior; M, middle; S, superior; RTA, road traffic accident (Trauma) group; HC, healthy controls.*

**FIGURE 1:**
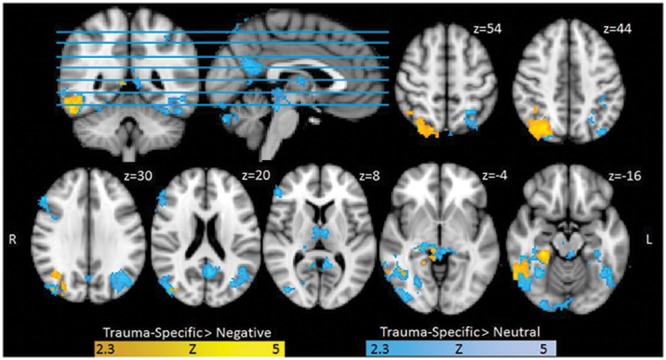
**Functional Activation for Trauma group RTA over healthy controls (HCs), Trauma-specific Stimuli > Neutral Stimuli, and Trauma-specific stimuli > Negative stimuli.** Results are cluster corrected (*Z* > 2.3, *p* < 0.05) and superimposed on the Montreal Neurological Institute (MNI; 2 mm) template brain. Coordinates are shown in mm (z-direction) of the MNI template brain.

Exploring Trauma-specific stimuli contrasted with Neutral stimuli, we found higher activation for trauma exposed participants in several clusters including, bilateral superior and inferior occipital cortex, bilateratal inferior temporal gyrus and temporal fusiform areas, left precuneus, bilateral posterior cingulate gyrus, right thalamus and lingual gyrus, right frontal areas, right thalamus and hippocampal areas. When contrasting Trauma-specific vs. Negative stimuli, we found higher activation for trauma exposed participants in right lateralized occipital and temporal areas, as well as posterior cingulate gyrus and parahippocampal areas (see **Figure 1**). We found no between group differences for Negative stimuli contrasted with Trauma-specific stimuli or Neutral stimuli.

**FIGURE 2:**
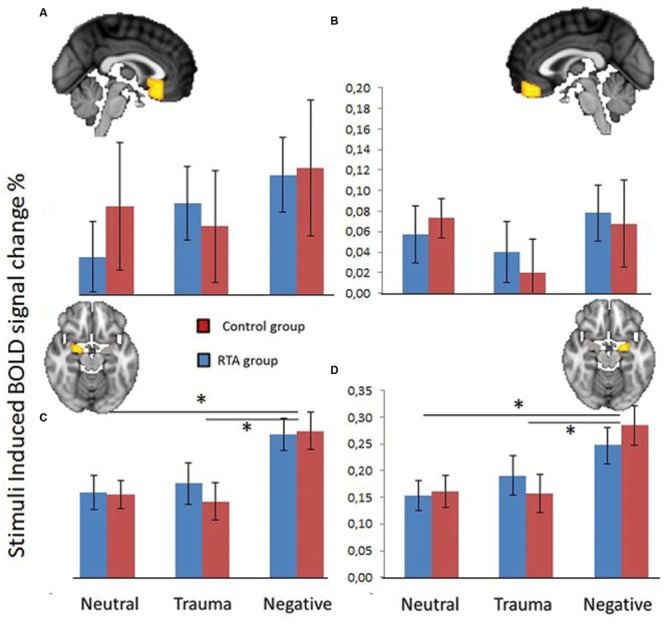
**Blood oxygen level dependent (BOLD) signal change (%) in subcallosal cortex (A), fronto medial cortex (B), left amygdala (C), and right amygdala (D), over three stimuli conditions; Neutral, Trauma-specific, Negative.** Blue equals Trauma group (RTA), and red equals HCs. Error bars represent 1 standard error of the mean. *p < 0.05.

Data from the four ROIs (left and right amygdala, subcallosal cortex, mPFC) revealed a significant main effect of stimuli type (Neutral, Trauma-specific, Negative) on mean percent signal change *F*(8,30) = 6.77, *p* < 0.0001, $\eta_{p}^{2}=0.643$. There were no significant main effect of group *F*(4,34) = 0.075, *p* = 0.989, $\eta_{p}^{2}=0.009$ or significant interaction between group and stimuli condition *F*(8,30) = 0.568, *p* = 0.796, $\eta_{p}^{2}=0.131$. Follow up univariate tests showed a significant linear effect for the right amygdala *F*(2,74) = 13.656, *p* < 0.0001, $\eta_{p}^{2}=0.27$, and a significant effect in the left amygdala *F*(2,74) = 16.854, *p* < 0.0001, $\eta_{p}^{2}=0.313$. Follow up pairwise comparisons (Bonferroni corrected) show that the mean percent signal change for the Negative stimuli condition was significantly higher than both the Neutral and Trauma-specific stimuli conditions, in the left (Mean Difference; *MD* = 0.114, *SE* = 0.020, *p* < 0.001 and *MD* = 0.112, *SE* = 0.024, *p* < 0.001, respectively) and right amygdala (*MD* = 0.11, *SE* = 0.020, *p* < 0.001 and *MD* = 0.094, *SE* = 0.022, *p* < 0.001, respectively; **Figure 2**).

A follow up repeated measures ANOVA with group as between subjects variable and PCL scores as covariate revealed no significant effects of PCL scores on percent BOLD signal change. A separate analysis on the linear relationships between PCL scores and any of the four ROIs for any of the stimuli conditions, revealed no significant correlations.

**FIGURE 3:**
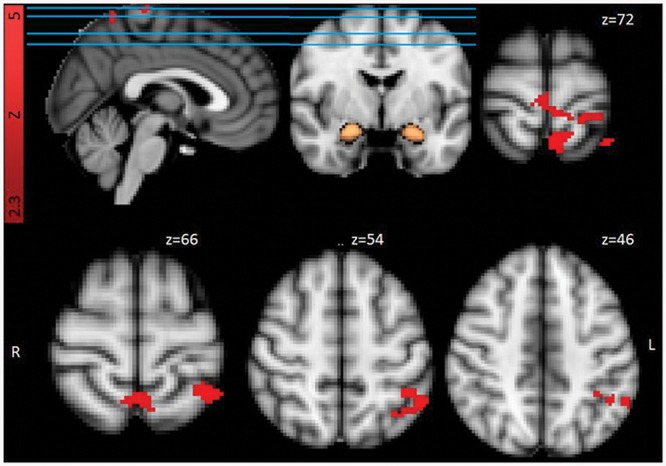
**Functional connectivity from bilateral amygdala mask (in copper) for Trauma group (RTA) over HCs, Trauma-specific Stimuli > Neutral Stimuli.** Results are cluster corrected (*Z* > 2.3, *p* < 0.05) and superimposed on the MNI (2 mm) template brain. Coordinates are shown in mm (z-direction) of the MNI template brain.

For the functional connectivity (PPI) analyses (see **Table 3** and **Figure 3**) results indicated higher functional connectivity in response to Trauma-specific stimuli for the RTA group compared to controls, between bilateral amygdala and three clusters including, bilateral supramarginal gyrus, angular gyrus, superior parietal and occipital areas, right lateralized temporal areas, and left lateralized post-central gyrus and frontal pole areas. We observed lower functional connectivity in response to neutral stimuli for the RTA group, between bilateral amygdala and bilateral supramarginal gyrus, right lateralized precentral, post-central and angular gyrus, left lateralized superior parietal areas, insular and frontal pole areas, and the central opercular cortex.

**Table 3:** Measured difference in functional connectivity, with bilateral amygdala as seed.

Voxels	*p*	*Z*	*x*	*y*	*z*	max *Δr*	Side		Peak location

**Neutral > baseline, HC > RTA**									
1190	<0.000001	3,85	–42	–48	62	0,31	L	34%	S. Parietal Lobe
		3,63	–62	–34	36		L	76%	Supramarginal G.
		3,37	–26	–54	44		L	36%	S. Parietal Lobe
		3,35	–46	–42	42		L	31%	Supramarginal G.
604	0.000581	3,18	50	–40	42	0,244	R	46%	Supramarginal G.
		3,18	46	–12	56		R	46%	Precentral G.
								25%	Post-central G.
		3,12	50	–2	50		R	70%	Precentral G.
		3,11	56	–48	44		R	53%	Angular G.
		3,09	54	–42	46		R	58%	Supramarginal G.
432	0.00681	3,23	–30	20	6	0,323	L	47%	Insular C.
		3,19	–38	4	2		L	59%	Insular C.
		3,1	–46	4	6		L	57%	Central Opercular C.
349	0.0249	3,54	–38	40	14	0,275	L	47%	Frontal Pole
		3,46	–40	38	22		L	34%	Frontal Pole
								28%	M. Frontal G.
		3,09	–30	58	6		L	83%	Frontal Pole

**Trauma-specific > baseline, RTA > HC**									

822	<0.00001	4,06	56	–42	30	0,243	R	48%	Supramarginal G.
		3,84	58	–46	32		R	42%	Angular G.
		3,5	58	–42	16		R	41%	Supramarginal G.
		3,5	52	–38	8		R	32%	Supramarginal G.
								16%	S. Temporal G.
		3,14	52	–38	18		R	22%	Supramarginal G.
								10%	Planum Temporale
		3,12	42	–56	48		R	28%	Angular G.
								20%	L. Occipital C.
501	0.00191	3,69	–54	–44	50	0,279	L	57%	Supramarginal G.
		3,57	–46	–38	62		L	34%	Post-central G.
		3,34	–44	–52	54		L	31%	Angular G.
		3,29	–38	–58	56		L	27%	L. Occipital C.
		3,26	–44	–46	60		L	30%	S. Parietal Lobe
		3,09	–54	–44	44		L	47%	Supramarginal G.
328	0.0294	3,86	–28	50	16	0,249	L	71%	Frontal Pole
		3,08	–28	58	12		L	87%	Frontal Pole

**Trauma-specific > Neutral, RTA > HC**						**Mean *Δr***			

426	0.0113	3,66	–38	–50	62	0,666	L	54%	S. Pairetal Lobe
			–34	–50	66		L	72%	S. Pairetal Lobe
			–42	–44	54		L	34%	S. Pairetal Lobe
								19%	Supramarginal G.
380	0.0224	3,77	–4	–50	72	0,846	L	13%	Post-central G.
								12%	Precuneous C.
			–14	–36	78		L	52%	Post-central G.
			–12	–44	74		L	48%	Post-central G.
								15%	S. Pairetal Lobe
			–6	–36	72		L	35%	Post-central G.
								18%	Precentral G.
			4	–28	74		R	56%	Precentral G.

*Max and mean differential functional connectivity (PPI) for clusters surviving corrections and threshold. Peak coordinates are in MNI space (mm). Z scores are reported for each cluster peak. Cluster extension: peaks above 10% of ROIs as defined by the Harvard Cortical and Subcortical Probability Atlas (http://fsl.fmrib.ox.ac.uk/fsl/fslwiki/Atlases) are reported. Δr denotes max/mean change in correlation between bilateral amygdala seed and cluster, between groups, for the given contrast. L, lateral; C, cortex; G, gyrus; I, inferior; M, middle; S, superior; RTA, road traffic accident (Trauma) group; HC, healthy controls.*

For the contrast Trauma-specific over Neutral, we observed higher functional connectivity for the RTA group in, left lateralized superior parietal areas, precuneus, supramarginal and post-central gyrus, and bilateral precentral gyrus.

No other contrasts revealed significant clusters of differential functional connectivity.

## Discussion

In the present study we investigated processing of Trauma-specific vs. Negative, and Neutral stimuli in participants who had recently been involved in a RTA, compared with HCs. Based on previous studies with trauma-exposed individuals without PTSD (Patel et al., 2012), we predicted heightened amygdala response to Trauma-specific stimuli, and differences in mPFC activity between groups.

Whole brain analysis of the neuro-functional differences for the RTA group over HC revealed higher Trauma-specific activation in visual processing related areas, when contrasted with Neutral and Negative stimuli, including occipital and inferior temporal cortices, as well as posterior cingulate. The connectivity analyses showed higher Trauma-specific functional connectivity (for the RTA group, compared to Neutral stimuli) between the bilateral amygdala seed and superior parietal regions including somatosensory areas, as well as precuneus and precentral gyrus.

The predicted signature activations (for trauma exposed participants) in amygdala and medial prefrontal regions were not observed in the ROI or whole brain analysis. However, we did observe hyper activation of amygdala for Negative violence pictures compared with Trauma-specific and Neutral stimuli, across groups, suggesting an overall higher threat appraisal of the Negative stimuli, in both groups. However, this might be caused by the perceived valence or arousal of the photos. Even though arousal and valence were matched based on self-reports obtained from a previous pilot study by Ehlers and Clark (personal communication, 2011), the Negative Violence photos might have been perceived as more threatening in our sample, thus the higher amygdala activation. It is also important to note that while the RTA group had significantly higher PCL scores than HC, only four scored over a cutoff for possible future PTSD diagnosis. This might indicate that the participants did not experience the traffic accident as particularly traumatic or that they were not severely affected by it at the time of the study. Hence, we do not know if the results would have been different in a group reporting higher levels of traumatic stress.

The overall whole brain results might indicate an attentional bias for trauma-specific reminders, for trauma-exposed participants, reflected by an increase in functional activity in visual, sensory, memory, and attention related areas. In the PTSD literature, attentional bias for trauma-relevant stimuli is well-established (e.g., Williams et al., 1996; Buckley et al., 2000; Bar-Haim et al., 2007). Thus, the present results might suggest that a trauma specific attentional bias, or heightened vigilance, can be present early after trauma-exposure in individuals without PTSD. In a recent meta-analysis Sartory et al. (2013) noted that PTSD participants showed increased precuneus activity, relative to trauma exposed controls in trauma reminder tasks. They concluded that trauma-specific stimuli/reminders are internalized to a higher degree in trauma-exposed individuals who develop PTSD. While the present results did not show higher functional activity in the precuneus for Trauma-specific stimuli contrasted with Negative stimuli, we did observe higher activation compared to Neutral stimuli as well as higher functional connectivity between amygdala and precuneus for the same contrast, suggesting perhaps not a trauma specific but threat related bias. However, cluster uncorrected data indicate low power might be a factor. While Sartory et al. (2013) note that for PTSD groups, attention might be directed inward toward a elicited trauma memory, our results with trauma-exposed individuals shows a heightened activity in attentional and sensory association areas, suggesting a more external bias.

Participants were required to respond to a specific occasional target during stimulus presentation. As such, the results could be interpreted as differential anticipation of target during the different stimuli conditions. In such a view, the results indicate a heightened visual anticipatory state for the RTA group, influenced by Trauma-specific stimuli, in accordance with a visual attentional threat related hypothesis.

The results also showed higher functional connectivity between amygdala and somatosensory areas, during Trauma-specific stimulus presentation for the RTA group as compared to Neutral stimuli, areas also implicated in the meta-analyses by Patel et al. (2012) and Stark et al. (2015). This might reflect a tighter coupling between Trauma Specific sensory and emotional processing. A similar argument has been presented by Falconer et al. (2008) where they observed increased somatosensory processing, but decreased inhibitory control during an inhibition task, suggesting that this heightened sensory processing might be associated with a failure in top down regulation as in the fear circuitry model (Rauch et al., 2006). However, the present results indicated heightened activation in laterlized frontal regions for Trauma Specific stimuli contrasted with Neutral stimuli, which might indicate, along with Patel et al. (2012) suggestion, active top down control, although the results do not implicate medial prefrontal regions.

Future studies should investigate whether levels of post-traumatic stress are associated with increased functional connectivity between amygdala and somatosensory and attentional systems, shortly after a traumatic experience, and how connectivity and neuro-functional activity patterns develop over time. The classic fear circuitry model of PTSD (Rauch et al., 2006), posit a hyperactive amygdala and hypoactive mPFC regions, and such an activity pattern might arise when the connection between amygdala and trauma-specific attentional processing becomes strong enough, or are internalized. Indeed, in a PET study, Gilboa et al. (2012) found increased functional connectivity between amygdala and visual cortices for repeated trauma reminders, for PTSD participants compared to trauma exposed controls. Furthermore, Palombo et al. (2015) studied survivors of a near plane crash, and reported increased BOLD response for trauma-specific stimuli in the occipital lobe, precuneus, thalamus, hippocampus and parahippocampal areas, while Patel et al. (2012) also reported functional activation differences between trauma-exposed and non-exposed participants in several regions, including precuneus.

The present study has some strengths and limitations that should be mentioned. Differences in design, timing of measurement, and PTSD status of the participants, make comparisons with previous findings difficult. While most previous studies have primarily focused on neuro-functional alterations associated with PTSD, the present study focused on trauma-exposure *per se*, shortly after the traumatic event. In the present study, the two groups did differ significantly on the PCL, however, none of the participants had a clinical diagnosis of PTSD, and only four scored over a cutoff for possible PTSD on the PCL scale (all in the RTA group). The participants in the present study were scanned within 3 weeks after their individual traffic accidents, which is earlier than the 1 month criteria for PTSD diagnosis (APA, 2000). Levels of PTSD symptoms is dependent of timing of measurement (McFarlane, 1997), hence the early time-window and low grade of symptomology might explain why we didn’t see the predicted activations in amygdala nor the mPFC. In addition, we could have included other previously reported regions, like dACC (Hughes and Shin, 2011), in our ROI analysis, however the whole brain results did not indicate differential medial prefrontal activations. Furthermore, a wider range in PCL scores, along with a larger sample, would have enabled us to distinguish between patterns of brain activity associated with different levels in post-traumatic stress reactions.

The early time window and the homogeneity of type of trauma-exposure also add to the strengths of the present study. Even though the short time window makes comparisons with previous findings more difficult, we believe it’s a necessary piece in understanding functional changes in the brain following a traumatic experience. Furthermore, given the present results and previous studies (e.g., Patel et al., 2012), future studies should employ both a trauma-exposed and non-exposed control groups, to disentangle the effects of PTSD and trauma-exposure *per se*, and to increase generalizability of results.

## Conclusion

The present results suggest that experiencing a potentially traumatic event alone might not be sufficient to cause the predicted activity levels in amygdala and mPFC. However, we propose that experiencing a traumatic event might cause an increased attentional sensory processing bias toward trauma-specific reminders. Future studies should determine whether this attentional bias in turn can influence the development of persistent post-traumatic stress reactions. Furthermore, prospective longitudinal studies are needed to determine how patterns of functional connectivity and neuro-functional activity develop in relation to levels of post-traumatic stress reactions.

## Author Contributions

IB developed the research questions and fMRI paradigm and, collected the data, interpretation of the results and drafted the manuscript. AN collected the data, performed and interpreted the analyses and drafted the manuscript. SL participated in developing the research question and fMRI paradigm, consulted during data analyses and participated in the write-up of the study. ØE participated in developing the research question and participated in the write-up of the study. LS participated in developing the research question, coordinated recruitment of participants and participated in the write-up of the study. TE consulted during technical set up, data analyses, and participated in the write-up of the study. BØ participated in developing the research question and participated in the write-up of the study. TH participated in developing the research question and participated in the write-up of the study.

## Conflict of Interest Statement

The authors declare that the research was conducted in the absence of any commercial or financial relationships that could be construed as a potential conflict of interest.

## Footnotes

1 www.fmrib.ox.ac.uk/fsl

2 http://fsl.fmrib.ox.ac.uk/fsl/fslwiki/Atlases
